# Indirect tax accountability as record agreement: inventory accounting and auditing under digital technologies and big data

**DOI:** 10.3389/fdata.2026.1953628

**Published:** 2026-10-28

**Authors:** Nigora Alimkhanova, Rakhmonkul Hamidovich Djalilov, Salima Rayimovna Egamberdieva, Jasurbek Anvarovich Ismatullaev, Otabek Normamatovich Kholmuradov, Nafosat Oramova, Oman Khushmuradov, Lobarkhon Anvarova, Asqarjon Asrorovich Raimberdiev

**Affiliations:** 1 Department of Accounting and Auditing, Karshi State Technical University, Qarshi, Uzbekistan; 2 Department of Tourism and Marketing, Karshi State University, Qarshi, Uzbekistan; 3 University of Information Technology and Management, Qarshi, Uzbekistan; 4 Department of Uzbek Language and Literature, University of Economics and Pedagogy, Qarshi, Uzbekistan; 5 Department of Economics and the Economies of Foreign Countries, Turon University, Qarshi, Uzbekistan; 6 Department of Primary Education, Karshi State University, Qarshi, Uzbekistan

**Keywords:** big data, continuous auditing, electronic invoicing, indirect taxation, inventory accounting, tax compliance, Uzbekistan, value added tax

## Abstract

An industrial enterprise keeps three accounts of the same goods. Operations hold a physical record of what moved, the enterprise system holds a transactional record of what was documented and priced, and the tax function holds a fiscal record of what was declared. Indirect taxation works to the extent that the three records agree. The standard evasion schemes in manufacturing, namely unrecorded output, invented purchasing, disguised disposal and elastic valuation, all drive the records apart while leaving each one internally plausible. Serialized marking, sensor networks, enterprise event logs and clearance-model electronic invoicing have changed what can be tested, because the three records now exist at population scale and in machine-readable form. Drawing on tax compliance economics, big data research in auditing, continuous assurance and organizational capability theory, this article reconstructs indirect tax accountability as a property of agreement among the three records rather than of any one of them. It first states the conditions under which agreement is evidence at all. A reconciliation carries weight only where its two sides descend from causally separate processes, so the records must contain measurement anchors generated outside the enterprise transaction-processing layer. Integrated systems violate that condition by default. Agreement is then formalized as a set of reconciliation residuals, and a detection condition is derived from stated assumptions. A scheme survives only while its divergence stays inside the noise floor of the tightest independent check that sees it, so the sustainable tax gap is the sum of those noise floors rather than a function of the number of controls in place. A sensitivity analysis shows how the bound moves when the assumptions of separability, exogenous tolerances and zero detection risk are relaxed. Four leakage channels are traced through the inventory cycle, matched to the reconciliation that exposes each, and condensed into four propositions with refuting observations and candidate measures. Uzbekistan is the instantiating setting, where clearance invoicing, serialized labeling and, from January 2026, automated invoice risk scoring have outrun enterprise capability, and where documented administrative reconciliation practice between 2021 and 2023 supplies a live episode and its governance failure mode.

## 1 Introduction

On the last evening of a tax period the paperwork of an industrial plant is usually in order: invoices reconcile to the ledger, the ledger to the return, and the return will survive a desk check. The yard tells a different story. Pallets have moved without documents, a batch was written off before an inspection, and finished goods bear an uncertain relation to the materials that entered the gate. Both scenes describe the same enterprise. Which one the tax system sees is the subject of this paper.

The value added tax now anchors consumption taxation in most of the world (Ebrill et al., 2001). Its celebrated feature is built-in enforcement: each buyer claims credit for the tax its supplier charged, so the two sides of every transaction report on each other, and revenue depends on the integrity of that chain more than on the rate (Keen and Lockwood, 2010). Field evidence bears the design out. Enforcement letters in Chile deterred evasion least where transactions carried a documentary trail and most at the final sale where the trail ends (Pomeranz, 2015). A Danish experiment found evasion near zero under third-party reporting and substantial under self-reporting (Kleven et al., 2011). A Brazilian programme that turned consumers into receipt auditors lifted reported retail revenue by roughly a fifth (Naritomi, 2019).

The same experiments expose the design's soft spot. Firms do not stop misreporting when one margin becomes visible; they move to margins that remain unobserved. Ecuadorian firms raised reported costs almost in step once revenue became visible (Carrillo et al., 2017), and United States businesses offset newly visible card receipts elsewhere on the return (Slemrod et al., 2017). Enforcement is bounded by its least observable margin (Slemrod, 2019). For a manufacturer that margin is not on any form. It is the flow of goods through procurement, storage, production, sale and disposal, which accounting has always measured badly. One retailer's recorded quantity was wrong at about two thirds of item locations (DeHoratius and Raman, 2008).

What has changed is the cost of watching that flow. Decision making has shifted from samples to full populations (McAfee and Brynjolfsson, 2012; Chen et al., 2012), and the properties of big data now describe the ordinary output of factory sensors, marking systems and enterprise software (Gandomi and Haider, 2015). Accounting has absorbed part of the shift (Vasarhelyi et al., 2015) and administrations are building taxation into the systems firms already run (OECD, 2020). The returns are real but uneven: e-invoicing raised reported sales in Peru (Bellon et al., 2022), e-filing cut time and bribes in Tajikistan (Okunogbe and Pouliquen, 2022), and Italian e-invoicing is associated with a fall in cross-border fraud (Heinemann and Stiller, 2025). Rwandan evidence sharpens the point: e-invoicing raises payments mainly by making audits more efficient, not by making adopters honest (Kotsogiannis et al., 2025), and gains concentrate where surrounding processes are reorganized around the data (Hesami et al., 2024).

Read together, these results point to a question none of the underlying literatures asks. Compliance economics models the information available to the state, not the data architecture of the firm; big data auditing studies evidence for financial statements, not for tax; continuous assurance monitors business processes but has not reached the indirect tax cycle. The gap sits where evasion actually happens, inside the enterprise, between what the goods did and what the documents say.

This paper builds the missing connection. Its central claim can be stated in one sentence: indirect tax accountability is a property of agreement among three independently generated records of the same goods, and digital technologies matter for taxation because they make that agreement testable continuously, completely and cheaply. We should be candid at the outset about what in this claim is new and what is not. The practice of matching documents to goods and goods to declarations is as old as auditing itself; three-way matching is a textbook control, and no experienced auditor will find the triangle of Figure 1 unfamiliar. The contribution claimed here is not the triangle but what is built on it. First, the paper states the conditions under which agreement between records is evidence of anything. Integrated enterprise systems have undermined those conditions, and Section 4.4 makes them explicit. A reconciliation carries evidential weight only where its two sides descend from causally separate generating processes. Much of what passes for reconciliation inside a modern enterprise system compares a record with a copy of itself. Second, the familiar matching heuristics are converted into a formal detection condition with a derived bound, Equation 7. Its assumptions are stated, and its behavior when they are relaxed is analyzed in Section 4.5. The bound has comparative statics that can be wrong, which is what distinguishes a framework from a description. Third, the framework is instantiated in a jurisdiction that has actually operated a population-scale cross-record reconciliation with cash consequences, and Section 9.3 reads that documented episode, including its withdrawal, through the framework's own categories.

**Figure 1:**
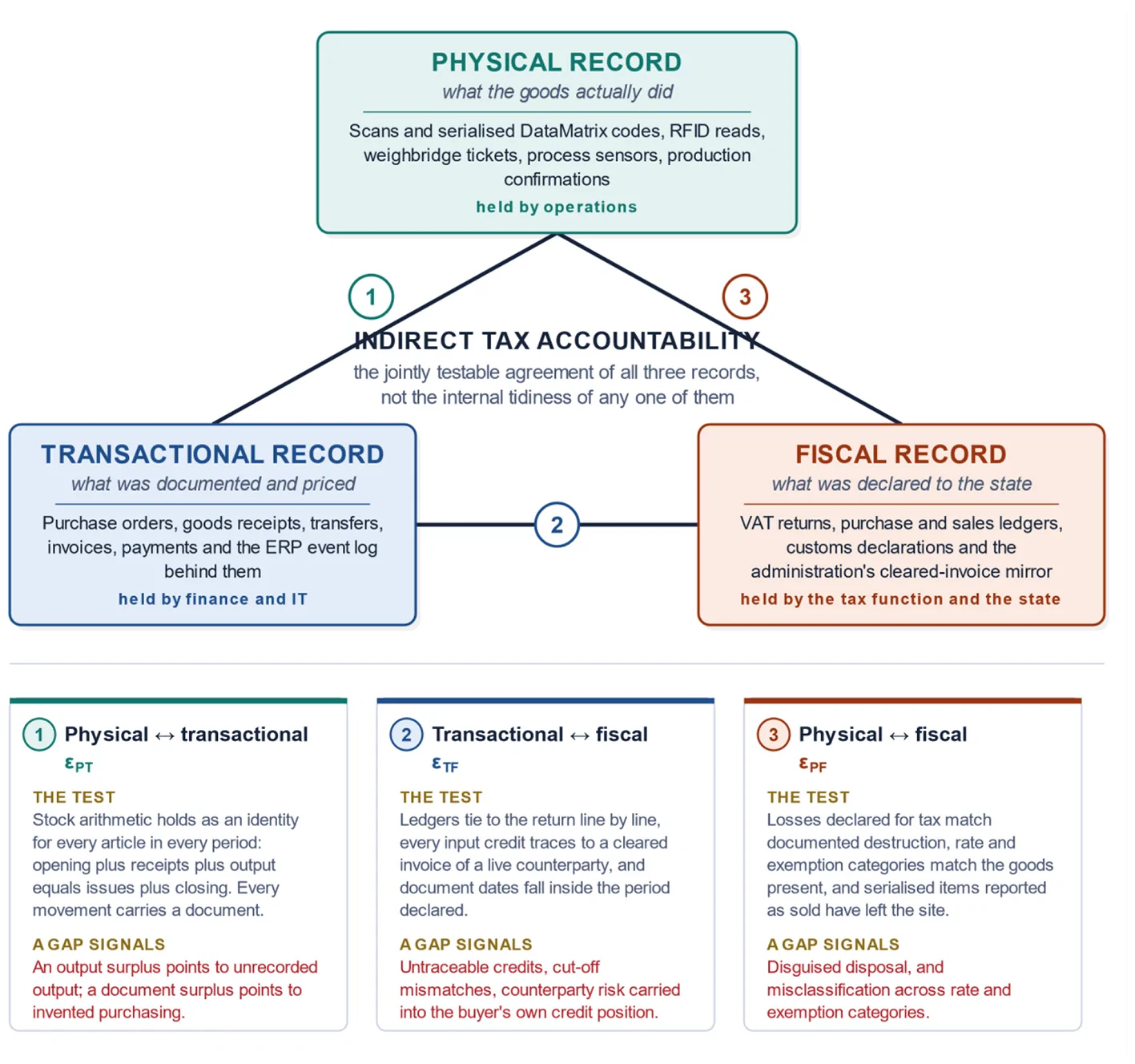
The record-agreement triangle. Three independently generated records of the same goods and the three pairwise reconciliations between them. Accountability is a property of the edges, not of the vertices.

Section 4 states the claim formally. It defines the three records, the residuals that measure their disagreement, the independence conditions under which those residuals are informative, and a detection condition. The principal result follows: the gap an enterprise can sustain is bounded by the sum of the measurement tolerances of the independent checks that bind. Sections 5 to 7 apply the framework to four leakage channels, the analytics that expose them and the deterrence they generate. Section 8 states four propositions with their mediation structure and the constructs by which each could be tested. Section 9 instantiates the framework in Uzbekistan, where the administration's own reconciliation practice between 2021 and 2023 supplies documented empirical material.

Three contributions follow. The first is a change of unit of analysis, from the reporting decision or the enforcement production function to the relations among an enterprise's own records, together with the independence conditions that make those relations evidential. The second is analytical: expressing accountability as residuals converts a set of familiar audit heuristics into a derived condition with determinate comparative statics and a stated domain of validity, which in turn explains why hardening one record while leaving the comparison loose buys nothing. The third is empirical groundwork. The propositions are stated with their refuting observations and with measures attached, and the framework is confronted with the one documented population-scale application of cross-record reconciliation in the instantiating jurisdiction. It is therefore exposed to evidence rather than protected from it.

## 2 Conceptual method and evidence base

### 2.1 Design

No new enterprise-level data are reported here. The paper practices theory synthesis, joining research streams that developed apart and showing the join carries explanatory weight (Jaakkola, 2020); its goals are integrative and generative (MacInnis, 2011). Construction followed the phases Jabareen (2009) prescribes: fields mapped, load-bearing concepts named, points of friction identified, and the synthesis revised until it absorbed the main regularities each field reports. The concept examined is indirect tax accountability, its elements are the three records, and the connections are the reconciliations of Section 4. Artifacts are offered under the design science standard of argued utility before field evaluation (Hevner et al., 2004).

### 2.2 Literature protocol

The literature base was assembled under an explicit protocol (Snyder, 2019). Scopus, Web of Science and Google Scholar were queried with combinations of tax terms (value added tax, compliance, enforcement, electronic invoicing), accounting terms (inventory, audit, internal control, information systems) and data terms (big data, analytics, process mining, continuous auditing). The citation networks of anchor papers were then followed in both directions. A source entered the base if it was refereed or issued by a standing international body, belonged to a field named in Section 3, and spoke to the accounting, auditing or taxation of goods flows. Abstracts without papers, unattributed gray material and engineering studies with no fiscal content stayed out. The review stands in one place: Section 3 carries it in full, and Sections 4 and 6 draw on it rather than opening literatures of their own.

### 2.3 Institutional evidence and its verification

Section 9 uses documentary evidence rather than microdata. Every legal instrument is identified by type, number and date, every quantitative statement is attributed to a named issuing body, primary publication is preferred to secondary summary, and amendments are stated. This matters: much of the descriptive literature on Uzbek tax digitalisation still cites Resolution No. 737 of 20 November 2020 as governing digital labeling, although Resolution No. 833 of 31 December 2020 superseded it 6 weeks later and sets the operating rules.

### 2.4 Scope

The framework addresses indirect taxes on goods, and value added tax in particular, in enterprises that transform materials into products. Services, financial supplies and pure resale lie outside it, not because the logic fails there but because the physical record thins until the central comparison loses power. Section 9.6 states these boundaries as conditions, since the framework's predictions change across them.

## 3 Theoretical background: the source literatures and the gap between them

### 3.1 The economics of tax compliance

The first stream descends from the model in which a taxpayer weighs concealment against the probability and price of detection (Allingham and Sandmo, 1972). Its findings are consistent: reported behavior tracks observability, third-party information beats self-reporting by an order of magnitude, and enforcement diverts misreporting rather than eliminating it (Carrillo et al., 2017; Kleven et al., 2011; Slemrod, 2019). It also treats enforcement design as an optimisation with costs (Keen and Slemrod, 2017). What it does not model is the firm's internal data landscape: the taxpayer is a reporting decision, not an organization with warehouses, event logs and master data, and the margin that bounds enforcement is a primitive rather than something a firm's systems could make observable.

### 3.2 Big data in accounting and auditing

The second stream has documented how population-scale records alter accounting information (Bhimani and Willcocks, 2014; Warren et al., 2015). It has shown how audit evidence expands to data whose provenance must be established (Yoon et al., 2015), and how procedures move from ratios to whole-population testing (Cao et al., 2015; Appelbaum et al., 2017; Gepp et al., 2018). It is equally frank about the friction. Sampling-era standards strain under populations (Alles, 2015; Krahel and Titera, 2015) and judgement degrades under signal volume (Brown-Liburd et al., 2015). Automation reorders the profession's tasks (Issa et al., 2016; Kokina and Davenport, 2017; Moll and Yigitbasioglu, 2019), and delivered accountability can fall short of advertised (Arnaboldi et al., 2017). Taxation barely appears as an object of assurance.

### 3.3 Continuous assurance

The third stream demonstrated three decades ago that auditing can run alongside the systems it audits (Vasarhelyi and Halper, 1991), and specified the capability required (Rezaee et al., 2002). Later work proved feasibility in a live environment (Alles et al., 2006) and mapped the passage to practice (Chan and Vasarhelyi, 2011). Most usefully, it wrote the arithmetic of business flows as continuity equations whose violation raises an alert (Kogan et al., 2014). Inventory is where such equations live most naturally, since stock arithmetic is an identity, yet the stream has aimed at financial reporting, not at the tax base.

### 3.4 Capability and institutional theory

The fourth stream answers why identical systems perform unequally. Advantage rests on resources competitors cannot copy (Barney, 1991) and on the capacity to sense, seize and reconfigure (Teece et al., 1997; Teece, 2007). Institutional analysis warns that structures adopted under outside pressure are often ceremonial (Meyer and Rowan, 1977), spread by coercion and imitation rather than usefulness (DiMaggio and Powell, 1983), and that societies can settle for rituals of checking (Power, 1997). This stream supplies the paper's moderator but not the content of tax data work.

### 3.5 Digital traces and analytic technique families

Two further bodies of work supply the raw material the framework runs on, and they are gathered here so that the review stands in one place rather than being introduced piecemeal in later sections.

The first concerns the digital traces themselves. Item-level identification was shown early on to pay for itself by cutting the costs of misplacement, shrinkage and record error, with returns rising in the degree of process integration (Lee and Özer, 2007). The internet of things has since carried observation into transit, storage conditions and machine states across the supply chain (Ben-Daya et al., 2019). Supply chain research treats this data layer as the principal site of analytical value (Waller and Fawcett, 2013), provided the data are integrated across functions rather than merely possessed (Kache and Seuring, 2017). The same layer feeds the models that anticipate disruption and its propagation through networks (Ivanov et al., 2019). Where records must be tamper-evident across organizational boundaries, append-only ledger designs offer one route (Dai and Vasarhelyi, 2017), though the accounting literature is right to insist that no ledger technology certifies the truth of what was entered into it (Schmitz and Leoni, 2019).

The second concerns the technique families that read those traces. Process mining replays business events from their logs, and field work has shown that the replay surfaces control breaches invisible to conventional procedures (Jans et al., 2014). Pointed at procurement and disposal, it exposes receipts recorded before orders, documents created in retrospective bursts, and single users completing a separation of duties alone. Descriptive mining of internal records has been shown to reduce fraud exposure in a corporate setting (Jans et al., 2010), and the mining of adjustment entries reaches the journals through which write-offs and revaluations are booked (Debreceny and Gray, 2010). Digit analysis under Benford expectations screens whole populations of quantities and amounts for invention at negligible cost (Nigrini and Mittermaier, 1997). Supervised classifiers have outperformed traditional statistical screens in fraud detection benchmarks, with the standing caveat that rare-event imbalance and feature design govern their performance (Perols, 2011). For measurement questions the framework also draws on the methodology of formative index construction (Diamantopoulos and Winklhofer, 2001) and on the modern treatment of mediation analysis (Zhao et al., 2010), both of which become load-bearing in Sections 4.2 and 8.

### 3.6 The gap at the intersection

Table 1 sets the four streams side by side. Each answers its own question well. The question none answers, how a manufacturing firm can arrange its goods data so that its declarations become continuously verifiable, sits at their intersection, and that is where the mechanism lives. The compliance literature's unobserved margin, the auditing literature's population evidence, the assurance literature's continuity equations and the capability literature's implementation problem describe one object: the relation between an enterprise's records of its own goods.

**Table 1:** Four research streams, their central result, and what each leaves out.

Stream	Question it answers	Central empirical or theoretical result	What it leaves out	Anchor works
Economics of tax compliance	When do taxpayers report honestly?	Reporting tracks observability; third-party information dominates self-reporting; enforcement displaces misreporting to unobserved margins	The taxpayer's internal data architecture	Allingham and Sandmo (1972); Kleven et al. (2011); Carrillo et al. (2017); Slemrod (2019)
Big data in accounting and auditing	What do population-scale records do to evidence?	Full-population testing displaces sampling, at the cost of provenance, standards and judgement problems	Taxation as an object of assurance	Vasarhelyi et al. (2015); Cao et al. (2015); Appelbaum et al. (2017); Brown-Liburd et al. (2015)
Continuous assurance	When should testing happen?	Monitoring can run inside transaction processing, with continuity equations as the test logic	The indirect tax cycle	Vasarhelyi and Halper (1991); Alles et al. (2006); Kogan et al. (2014)
Capability and institutional theory	Why do identical systems perform unequally?	Capability and internalization decide whether adopted structures work or merely exist	The specific content of tax data work	Barney (1991); Teece (2007); Meyer and Rowan (1977); Power (1997)

The intersection of the four cells in the fourth column is the object of this paper.

## 4 The record-agreement framework

### 4.1 Three records of the same goods

Consider what an industrial enterprise knows about its goods. There is, first, a physical record: the observations produced by barcode scans, serialized codes, radio frequency tags, weighbridges and process sensors as materials and products move. It has grown dense and cheap, for the reasons reviewed in Section 3.5.

There is, second, a transactional record: the purchase orders, goods receipts, production confirmations, transfers, invoices and payments the enterprise system holds, each stamped with a time and a user. It prices the physical record and remembers the sequence in which things were documented, which is not always the sequence in which they happened. There is, third, a fiscal record: the returns, ledgers and customs declarations submitted to the state, with the mirror the administration keeps where invoices clear through its platform.

Figure 1 places the three records at the vertices of a triangle and the comparisons on its edges. Indirect tax accountability resides in the relations among the three records, not in any one of them. A fiscal record can be internally flawless while describing goods that never existed; a transactional record can be complete while omitting everything that moved without paper; a physical record can be precise while carrying no prices and no tax. Only their agreement is hard to fake, because the records are generated by different mechanisms with different owners, so a scheme must corrupt all three consistently rather than perfect any one.

### 4.2 Residuals: a formal statement

Stating the account precisely is what makes it testable. Let i index articles in the item master and t index tax periods. For each article and period the physical, transactional and fiscal records each imply a quantity moved and a tax due, denoted by the superscripts P, T and F.

The first relation is an identity rather than an estimate. For any article in any period, the opening balance plus receipts plus manufactured output equals issues plus write-offs plus the closing balance:


$$\begin{array}{l} S_{i,t-1}+R_{it}+M_{it}=U_{it}+W_{it}+S_{it\;} \end{array}$$ (1)

Equation 1 holds in the physical and transactional records separately, because it is arithmetic rather than accounting policy. It is the continuity equation of Kogan et al. (2014) where continuity is exact. The first residual, Equation 2, is the difference between the net movement the two records report:


$$\begin{array}{l} \varepsilon_{it}^{PT}=q_{it}^{P}-q_{it}^{T} \end{array}$$ (2)

The second residual, Equation 3, compares the tax the documents imply with the tax the enterprise declared:


$$\begin{array}{l} \varepsilon_{it}^{TF}=\tau_{it}^{T}-\tau_{it}^{F} \end{array}$$ (3)

The third, Equation 4, closes the triangle, comparing the tax implied by the physical flow, valued and classified as it should be, with the tax declared:


$$\begin{array}{l} \varepsilon_{it}^{PF}=\tau \left(q_{it}^{P},c_{i},p_{i}\right)-\tau_{it}^{F} \end{array}$$ (4)

where *c*_*i*_ is the article's correct rate or exemption category and *p*_*i*_ its correct base. Perfect agreement is the joint condition that all three residuals vanish for all articles and periods. A single index of agreement for enterprise j in period t follows by scaling each residual on a base of comparable size and weighting:


$$\begin{array}{l} A_{jt}=1-\Sigma_{k}w_{k}|\varepsilon_{jt}^{k}|/B_{jt}^{k},k\epsilon \left\{PT,TF,PF\right\} \end{array}$$ (5)

with B(PT) the total documented movement, B(TF) and B(PF) the declared tax, and weights summing to one. Three clarifications define the status of Equation 5. First, nothing downstream depends on it. The detection condition of Section 4.5, the channel typology of Section 5 and the propositions of Section 8 all operate on the raw residuals of Equations 2–4. Equation 5 exists only to summarize those residuals for reporting and for cross-enterprise comparison. Second, its functional form is a deliberate choice rather than an arbitrary one. It is a weighted L1 aggregation of scaled residuals, the standard structure of a formative index, in which the indicators cause the construct rather than reflect it. The psychometric machinery of reflective scales, internal consistency and factor loadings, therefore does not apply. The appropriate route is the one prescribed for formative measurement: content specification, indicator collinearity checks and criterion validity against external outcomes (Diamantopoulos and Winklhofer, 2001). Third, the weights are undetermined by theory, and we say so rather than hiding it. A defensible default sets them proportional to the tax at risk on each edge, w_k_ ∝ Σ_i_ r_i_ p_i_ for the articles the edge covers, so that the index penalizes disagreement where money is exposed. The criterion against which any weighting should ultimately be calibrated is audit-assessed additional liability, as Section 11.4 specifies. Equation 5 is therefore offered as a starting operationalisation and not as a validated instrument, and any empirical application should treat its weights as parameters to be estimated, not constants to be assumed.

### 4.3 Why agreement rather than accuracy

The framework does not say that each record should be accurate, which is the ordinary language of internal control and cannot be tested from inside the enterprise, since accuracy is a relation between a record and a world no other record observes. It says the records should agree, which can be tested, because each is generated by a different mechanism under a different owner. Auditing a single record for internal coherence certifies exactly the property a competent scheme preserves: every classic evasion pattern in manufacturing is a controlled divergence between two records, engineered to stay below the resolution at which anyone compares them. Raising that resolution is what digital infrastructure has made cheap, and it is the only thing that reaches the scheme.

An obvious objection deserves a direct answer: agreement between records does not guarantee accuracy, because three records can align while all being wrong, either because they share a bias or because they were manipulated together. The objection is correct, and the framework's claim must therefore be stated with care. Agreement is not proposed as proof of compliance; it is proposed as evidence whose strength is exactly the improbability of the records agreeing by any route other than the truth. That improbability is not a constant. It depends on how the records are generated, and it collapses to zero when the records descend from a common source. The claim therefore has to be conditional. Agreement among the three records is evidence of compliance to the degree that the records are generated by causally separate processes under separate control. Producing a consistent false account then requires coordinating manipulations across mechanisms and owners rather than editing one database. Section 4.4 states these conditions explicitly, shows why modern integrated enterprise systems violate them by default, and identifies the record components that restore them. Under those conditions the epistemic position is the standard one of triangulated evidence. Agreement raises the probability of compliance without certifying it, and coordinated fraud across independent mechanisms remains possible but costly. The framework prices that cost through the detection condition of Section 4.5: a coordinated scheme must hold every observable divergence inside every binding tolerance simultaneously and indefinitely. What the framework rules out is cheap fraud, not conceivable fraud. A control that raises the price of a scheme from editing one field to synchronizing three mechanisms, one of them held by the state, has raised the cost of evasion substantially, even though it proves nothing with certainty.

### 4.4 Independence: when agreement is evidence and when it is an echo

The previous section's conditional needs content, because the direction of enterprise software development runs against it. Modern integrated systems interlink the three records by design. A goods receipt posted once in the warehouse module generates the stock movement, the accounting entry and the draft purchase document from a single user action, and a production confirmation writes the output quantity into inventory and into costing simultaneously. In a clearance jurisdiction the sales invoice that the enterprise system issues becomes, after validation, both the transactional record and the raw material of the fiscal one, from which the return can be pre-filled. This interdependence must be incorporated rather than assumed away: where two records are copies descended from one entry, the residual between them tests transcription and software correctness, not truth. A reconciliation of a record against its own echo can be perfect while everything it describes is false, because the falsehood was entered once and propagated faithfully.

The framework therefore needs an explicit distinction. Call a component of a record a measurement anchor if it originates in a mechanism that cannot be written or edited from the enterprise transaction-processing layer, and a derived component otherwise. Anchors include a weighbridge ticket generated by the instrument itself, a serialized DataMatrix scan reported to the state marking system, and metered energy and material draw from the process historian. So are a cycle count performed by staff organisationally separate from warehouse operations, and the administration's cleared-invoice mirror together with the counterparty's own copy of a document. Falsifying any of them requires acting on the world or on a system outside the firm's ledger, not on the ledger. A stock balance computed from posted documents, an accounting entry auto-generated from a receipt, and a return pre-filled from cleared invoices are derived: they inherit whatever their source contains.

The independence condition can then be stated. A reconciliation edge carries evidential weight for article i only if at least one of its two sides contains an anchor for that article. The set K(i) of checks that see the article, introduced formally in the next section, must therefore be restricted to edges satisfying this condition. The consequences are immediate and they sharpen rather than weaken the framework. First, the physical-to-transactional comparison is informative precisely where the physical side is instrumented. Where an article's physical record is itself compiled from the same postings as the transactional record, ε^PT^ is identically zero by construction. The article is then, for detection purposes, unseen, and its effective tolerance is unbounded. Second, the transactional-to-fiscal comparison retains independent content even under full integration, but from an unexpected direction. The clearance model places a copy of every invoice with the state and with the counterparty, so faking this edge requires collusion across organizational boundaries, which is the costly kind. The pre-filled return, by contrast, adds no independent content, since it is derived from the same mirror. Third, the framework's coverage argument becomes a statement about anchors rather than about data volume. An enterprise holding a large volume of derived data can still be unobserved. For exactly this reason the maturity path of Section 10 begins with the binding of anchors, codes, scans, meters and counts, to the item master before any analytics are bought.

Two design implications follow for practice. Segregation of duties, a long-established control, reappears at the level of data generation. The value of a cycle count comes from who performs it, and the value of a marking scan comes from the state system's copy being append-only from the firm's point of view. And redundancy should be bought across mechanisms rather than within them: a second document-derived stock report adds nothing, while a single energy meter bound into the yield comparison adds an anchor that no ledger edit can reach. Section 9.4 translates both implications into the concrete configuration of the enterprise software actually in daily use in the instantiating jurisdiction.

### 4.5 Detection thresholds and the sustainable gap: derivation, assumptions and sensitivity

The residuals of Section 4.2 are never exactly zero in a working plant. Yield varies within engineering tolerance, weighbridges have precision limits, scanning is imperfect, and honest timing differences arise at every period end. Each comparison therefore carries a noise floor, a tolerance below which a divergence cannot be distinguished from ordinary variation. Write that tolerance for check k applied to article i in period t as θ_k, it_, expressed in units of the quantity moved.

The derivation of the bound below is set out explicitly, together with its assumptions and their consequences when relaxed, because a bound whose assumptions are hidden cannot be argued with. Four assumptions carry the derivation.

Assumption A1 (anchored checks). Every check in K(i) satisfies the independence condition of Section 4.4; checks that compare a record with its own echo are excluded from K(i). Assumption A2 (separability). Detection operates article by article: a divergence engineered in article i moves only the residuals of article i, and checks bind at the article level rather than only at some aggregate. Assumption A3 (known, exogenous tolerances). The tolerances θ_k, it_ are fixed properties of the instrumentation, are not chosen by the evader, and are known to the evader, which is the conservative case, since an evader ignorant of the tolerances can only do worse. Assumption A4 (zero-detection strategy). The evader sustains the scheme indefinitely and accepts no positive probability of generating a flagged residual; ‘sustainable' means exactly this.

Under these assumptions the argument runs in three steps. A divergence of size δ engineered in article i is exposed by check k if and only if it exceeds that check's tolerance, as stated in Equation 6:


$$\begin{array}{l} check\;k\;detects\delta \Leftrightarrow |\delta |>\;\theta_{k,it} \end{array}$$ (6)

Let K(i) be the set of anchored checks that see article i at all. Which checks these are depends on what has been instrumented: a marked product group is seen by all three edges, while an unmarked bulk input may be seen only by the physical-to-transactional comparison. An article with no anchor at all is seen by none, its effective tolerance is unbounded, and it contributes without limit to the gap, which is the formal counterpart of the coverage argument. The divergences that survive are exactly those that fit inside the tolerance of every check that sees them, so the binding tolerance for the article is the smallest of them, $\theta_{\text{it}}^{*}$ = min_k∈K(i)_ θ_k, it_. By A4 the evader's feasible set in article *i* is the interval |δ| ≤ $\theta_{\text{it}}^{*}$, and by A3 the evader can select its supremum. By A2 the choices across articles are unconstrained by one another, so the enterprise-level maximum is the sum of the article-level maxima, each valued at its tax consequence. Summing the tax at risk over articles gives the gap the enterprise can sustain through its inventory cycle without generating a single detectable residual:


$$\begin{array}{l} G_{jt}^{*}=\Sigma_{i}r_{i}p_{i}\theta_{it}^{*} \end{array}$$ (7)

where *r*(i) is the applicable rate and *p*(i) the unit base. Equation 7 is deliberately austere, and three consequences follow from it directly.

The first is that assurance investment pays only where it lowers a binding tolerance. Adding a fourth control that duplicates a comparison already tighter than θ^*^ leaves G^*^ unchanged, which is the formal version of a complaint auditors make informally about control inflation. The second is that strengthening one record beyond the others yields little, because θ^*^ is set by a comparison and not by a record: a perfect fiscal record compared against a loosely instrumented physical record yields a loose θPF. Investment belongs at the weakest comparison, not the weakest record. The third is that coverage matters as much as precision. Enlarging *K*(i), by instrumenting a product family that no check currently sees, can cut θ^*^ it discontinuously, whereas refining an already binding measurement yields smooth and diminishing returns.

A stylised illustration fixes the magnitudes. Suppose a plant produces 100,000 units of one article in a period at a base of 100 currency units, taxed at 12 percent, and the physical-to-transactional comparison binds because material yield legitimately varies by up to 2 percent. Equation 7 puts the sustainable gap at 0.12 x 100 x 2,000, that is 24,000 currency units per period, available without triggering any residual. Binding serialized marking events and energy draw into the same comparison narrows the tolerance to 0.5 percent and the gap to 6,000. Nothing here required a larger audit budget, a higher frequency or a change in the law; it required a tighter comparison. Hardening the ledger-to-return reconciliation instead, already an identity with a tolerance near zero, would change nothing, because that check was not binding. The parameters are illustrative and drawn from no enterprise; their role is to show that the framework generates magnitudes rather than exhortations.

Sensitivity of the bound to its assumptions. Each assumption can be relaxed, and stating what happens is more informative than defending the assumptions.

Relaxing A2 (separability). Where a check binds only at an aggregate, say a product family whose members share a mass balance, the evader can mask divergences against one another inside the family, and the relevant tolerance becomes the family-level one applied to the family total. The family tolerance is at least as large as any member's, and the masked divergences net out below it. The sustainable gap under aggregation therefore weakly exceeds the article-level bound: G^*^ computed at the finest keyed level is a lower envelope, and every coarsening of the item master raises it. This converts a data-management commonplace, that master data granularity matters, into a determinate prediction: enterprises that key their checks at family level should sustain larger measured gaps than enterprises keyed at article level with the same instruments, and the difference should grow in within-family heterogeneity.

Relaxing A3 (exogenous tolerances). A determined scheme designer can work to widen the tolerances themselves, for example by introducing genuine process variability that enlarges the legitimate yield band, or by degrading scan discipline so that the noise floor of the marking comparison rises. Under endogenous θ the bound still holds at each date, but θ^*^ becomes a choice variable and G^*^ rises linearly in it. Two further predictions follow. Variance itself becomes an object of manipulation and therefore of monitoring, so a second-moment control belongs in the architecture of Section 6, tracking the dispersion of yields and scan-match rates over time and not only their levels. And the comparative statics of investment are preserved in direction, since lowering a binding tolerance still lowers the bound one-for-one, though their magnitude is attenuated by whatever widening the designer can purchase.

Relaxing A4 (zero detection risk). An evader willing to accept a per-period probability α of generating a flagged residual can exceed θ^*^. Model the observed residual as the engineered divergence plus measurement noise, ε = δ + *u*, with the alert raised when |ε| exceeds the tolerance. The evader's feasible divergence at risk level α is the largest δ for which the alert probability stays at α. It exceeds θ^*^ by a margin that grows in the noise dispersion and in α, and the corresponding gap G^*^(α) is increasing in α, with G^*^(0) equal to Equation 7. Equation 7 is therefore the zero-hazard ceiling, a lower envelope on what a risk-tolerant evader can extract. Two things keep the envelope useful. The expected number of periods before a flag is 1/α, so the value of exceeding the ceiling is a wasting asset whose life shortens as comparison becomes more frequent. This is the formal content of Section 7's claim that continuous comparison changes the decision problem and not merely the audit calendar. And the excess over θ^*^ is priced in units of the noise dispersion, so the same investments that lower θ^*^ also shrink the risk-tolerant evader's headroom. Table 2 works the stylised example through both the deterministic tolerances and the aggregation relaxation to show the orders of magnitude involved.

**Table 2:** Sensitivity of the sustainable-gap bound of Equation 7 in the stylised example.

Scenario (stylised plant: 100,000 units per period, base 100, rate 12%)	Binding tolerance θ^*^	Sustainable gap G^*^, currency units per period	G^*^as share of output VAT (1,200,000)
Baseline yield tolerance, no additional anchors	2.0%	24,000	2.0%
Intermediate instrumentation	1.0%	12,000	1.0%
Marking events and energy draw bound into the comparison	0.5%	6,000	0.5%
Degraded scan discipline (endogenous θ, A3 relaxed)	3.0%	36,000	3.0%
Checks keyed at family level over heterogeneous articles (A2 relaxed)	family-level ≥ 2.0%	≥ 24,000, rising in within-family heterogeneity	≥ 2.0%

All figures follow deterministically from G^*^ = *r* × *p* × (θ^*^ × volume); the parameters are illustrative and are not drawn from any enterprise. Under relaxation of A4 every row becomes a lower envelope: an evader accepting per-period detection hazard α can exceed it, at the price of an expected 1/α periods before a flag.

## 5 Where the base leaks: four divergence channels

Four divergence patterns account for most of the indirect tax lost through inventory. Each attacks a specific joint between records at a specific stage of the cycle and moves a specific residual. Figure 2 places them along the cycle and links each to the reconciliation that closes on it.

**Figure 2:**
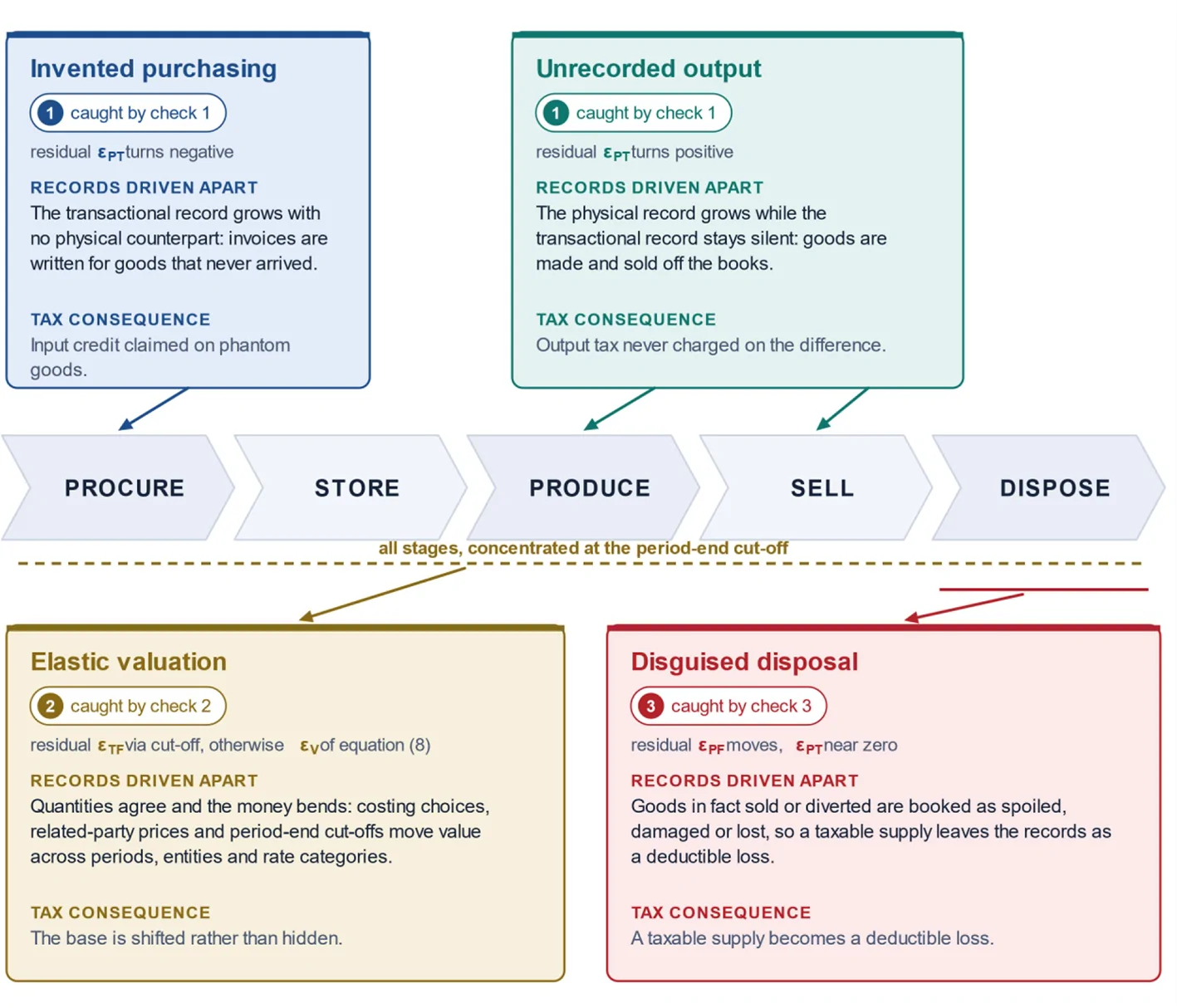
The four divergence channels and the stages of the inventory cycle they attack. Each channel carries the reconciliation of Figure 1 that closes on it and the residual of Section 4.2 that it moves. Elastic valuation reaches the transactional-to-fiscal comparison through cut-off timing only; its valuation component requires the benchmark test of Equation 8.

### 5.1 Unrecorded output

Goods are made and sold, the physical record grows, and the transactional record is simply not written, so output invoiced falls short of output produced. The quantity residual ε^PT^ goes positive while the tax residuals stay quiet, because the fiscal record faithfully reports a transactional record that is itself incomplete. This channel is the most sensitive to instrumentation: an enterprise that meters material and energy input can bound output from below independently of anything the sales system says.

### 5.2 Invented purchasing

The same joint is attacked from the opposite direction at procurement. Invoices are written for goods that never arrived, so the transactional record grows without a physical counterpart and the credit on the phantom purchase is set against real liabilities. Here ε^PT^ goes negative, and a second signature appears in the fiscal record, since phantom invoices tend to originate from counterparties that are dormant, newly registered or connected in circular flows. The channel sits across two comparisons, which is why it responds to warehouse instrumentation and to counterparty analytics alike, and why administrations that score invoices from outside the firm reach it first.

### 5.3 Disguised disposal

Goods that were in fact sold or diverted are recorded as spoiled, damaged or lost, converting a taxable supply into a deductible loss. Quantities are consistent between the physical and transactional records, so ε^PT^ stays near zero and the scheme is invisible to the comparison that catches the first two channels. It moves ε^PF^ instead: the tax implied by the physical exit of goods, which was a supply, differs from the tax declared, which recorded a loss. Its statistical fingerprint is temporal, namely the clustering of write-offs shortly before stocktakes and inspections, and its documentary fingerprint is the absence of destruction evidence proportionate to the loss claimed.

### 5.4 Elastic valuation, and the limit of the triangle

The fourth channel leaves quantities honest and bends the money. Costing methods, transfer prices to related parties and period-end cut-offs are chosen so that value, and with it tax, migrates across periods, entities or rate categories. This channel exposes a boundary of the framework rather than an application of it. All three residuals of Section 4.2 are anchored in quantity or in the tax that quantity implies, so a manipulation preserving quantity can hold ε^PT^ at zero while moving the base. Part of the effect reaches ε^TF^ through cut-off, since a document dated into the wrong period breaks the tie between ledger and return, and process mining of timestamps reads that directly. The valuation component reaches none of the three. It requires a fourth test of a different kind, a comparison of a record against an external expectation rather than against another record:


$$\begin{array}{l} \varepsilon_{it}^{V}=v_{it}^{T}-v_{it} \end{array}$$ (8)

where v is the value implied by an arm's length benchmark or by a driver-based cost model. Equation 8 is a within-record test with an external reference, and it inherits the weaknesses of any benchmark: it is only as good as the comparator, and it is contestable in a way that an arithmetic identity is not. We state this explicitly because a framework that claimed to close all four channels with three checks would be claiming too much. The triangle closes the quantity-based channels tightly and the valuation channel only partly, and enterprises that treat the two kinds of evidence as equally strong will find that out in a dispute.

## 6 Reading the residuals: analytics for the inventory tax cycle

### 6.1 Assigning technique families to channels

Comparison generates candidate gaps by the thousand; analytics decide which are noise, which are error and which are design. The technique families were reviewed in Section 3.5; what this section adds is the assignment. Table 3 assigns them to the four channels with the digital trace each consumes, the signature it seeks and the source of false positives that most often discredits it in practice.

**Table 3:** Analytics, digital traces and detection signatures, by divergence channel.

Channel	Principal technique family	Digital trace consumed	Signature sought	Principal source of false positives
Unrecorded output	Continuity and mass-balance analysis	Production confirmations; material and energy draw; serialized marking events	Yields outside engineering tolerance; input-implied output above invoiced output	Legitimate yield variation, rework, scrap booked against standards
Invented purchasing	Counterparty network analytics with risk scoring	Cleared-invoice ledgers; supplier registries; payment trails	Credits from dormant or freshly registered counterparties; circular flows	Genuine new suppliers, group restructuring, intermediated logistics
Disguised disposal	Digit analysis and temporal outlier detection	Adjustment journals; stocktake results; inspection calendar	Departures from Benford expectations in write-offs; loss spikes ahead of counts	Seasonal shelf-life effects, genuine incident clustering, policy-driven clean-ups
Elastic valuation	Driver-based prediction and price benchmarking; process mining of cut-offs	Costing module; market and related-party prices; document timestamps	Unit costs off their drivers; related-party prices off benchmarks; reversed event sequences at period end	Input price volatility, product mix change, legitimate late invoicing

Calibration to enterprise data precedes any use in an audit programme. The last column is not a caveat but a design input: a technique whose false-positive driver is not modeled will be abandoned by its users within one or two cycles.

### 6.2 Three disciplines

Three disciplines keep the machinery honest, and Figure 3 places them as gates every candidate finding must pass. Provenance comes first: population data are evidence only once completeness and origin are established (Cao et al., 2015; Yoon et al., 2015). Proportion comes second: sampling-era methods must be reread for a world where exceptions arrive in bulk (Alles, 2015; Krahel and Titera, 2015), and the evaluator, whose judgement deteriorates under overload (Brown-Liburd et al., 2015), must receive ranked and contextualized alerts. Ownership comes third and is most often skipped: an alert without a named owner, a deadline and a recorded disposition is a dashboard entry, not a control.

**Figure 3:**
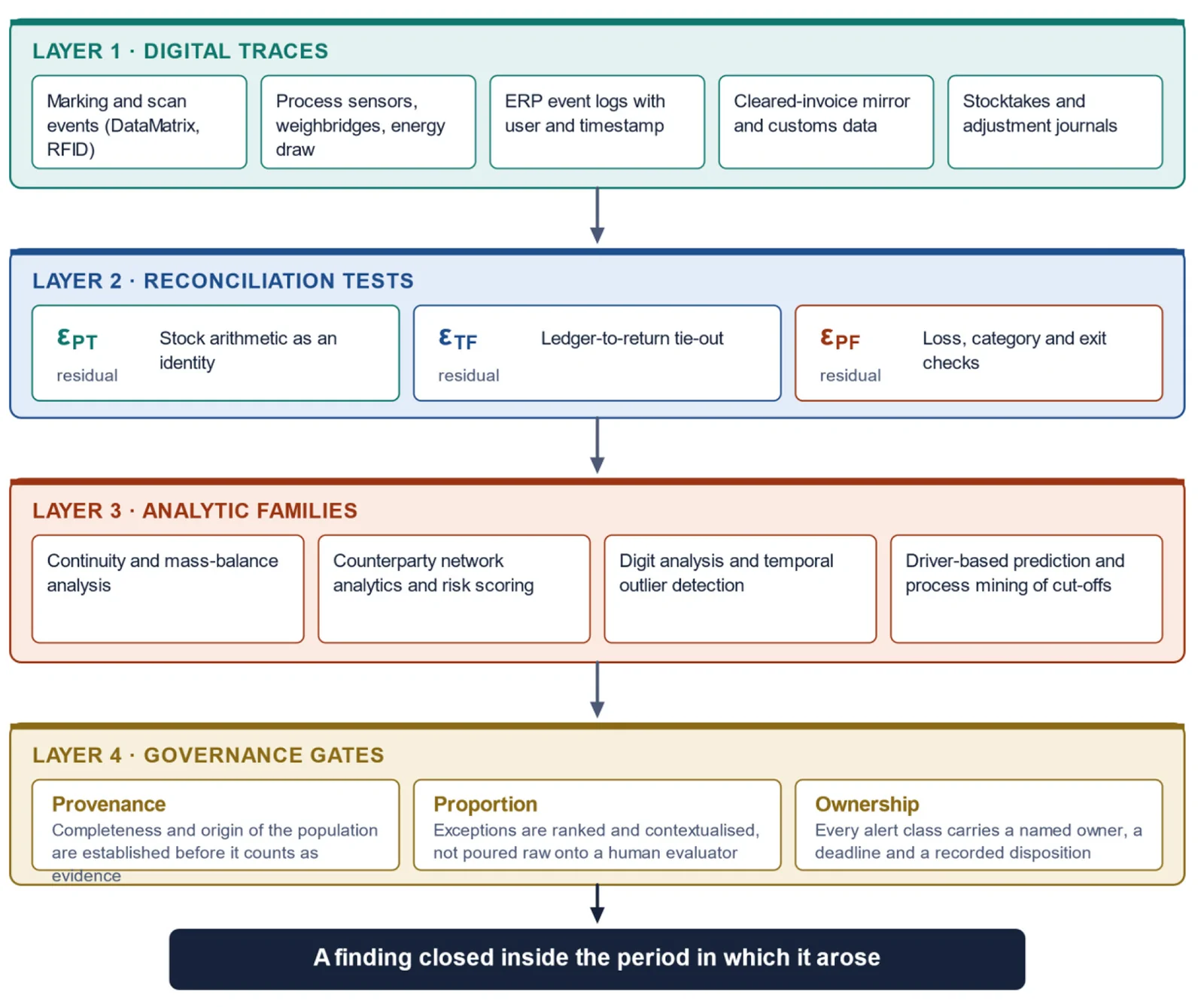
An analytics architecture for the inventory tax cycle. Digital traces feed the three reconciliations of Figure 1; the residuals feed technique families matched to the divergence channels of Figure 2; and every candidate finding passes three governance gates before it counts as a control.

## 7 Deterrence at the moment of decision

Detection is half the mechanism; the other half is what the prospect of detection does before anything is detected. In the deterrence tradition the decisive quantity is the probability of examination as perceived when a scheme is contemplated (Allingham and Sandmo, 1972), and compliance follows that perception closely (Kleven et al., 2011). Periodic audit concentrates examination into a brief season. Continuous comparison spreads it across every transaction, so the storekeeper booking a convenient write-off acts knowing the triangle will be checked this week rather than in 3 years.

Equation 7 sharpens this beyond the usual appeal to frequency. What continuous comparison changes is not only how often examination occurs but what the person contemplating a divergence must know to stay inside θ^*^. Under periodic audit the tolerance is set by a sample and unknown in advance, which makes small divergences look safe and occasionally large ones too. Under continuous comparison it is a published property of the enterprise's own instrumentation and binds every transaction. The scheme space contracts to a set whose boundary is stable and visible, a more uncomfortable situation than an unknown chance of a distant audit. The sensitivity analysis of Section 4.5 adds the complement: an evader who exceeds the boundary buys a wasting asset with expected life 1/α comparison periods, so shortening that period devalues risk-tolerant evasion in direct proportion.

The displacement results carry a lesson about coverage. Because visible margins push misreporting onto invisible ones (Carrillo et al., 2017; Slemrod et al., 2017), a regime watching sales alone would drive manipulation into purchasing, valuation and disposal. In the notation of Section 4.5 this is a statement about K(i): a check covering part of the cycle leaves the binding tolerance elsewhere untouched, and the gap migrates there intact. The external results on trails and rewards (Naritomi, 2019; Pomeranz, 2015) translate inward. The counterpart of the paper trail is the marking scan and the event log, and the counterpart of the consumer-auditor is the line owner who disposes of an alert under their own name.

## 8 Propositions and their measurement

### 8.1 Four propositions

The argument condenses into four statements, each paired with the observation that would defeat it. Figure 4 arranges them as a single model.

**Figure 4:**
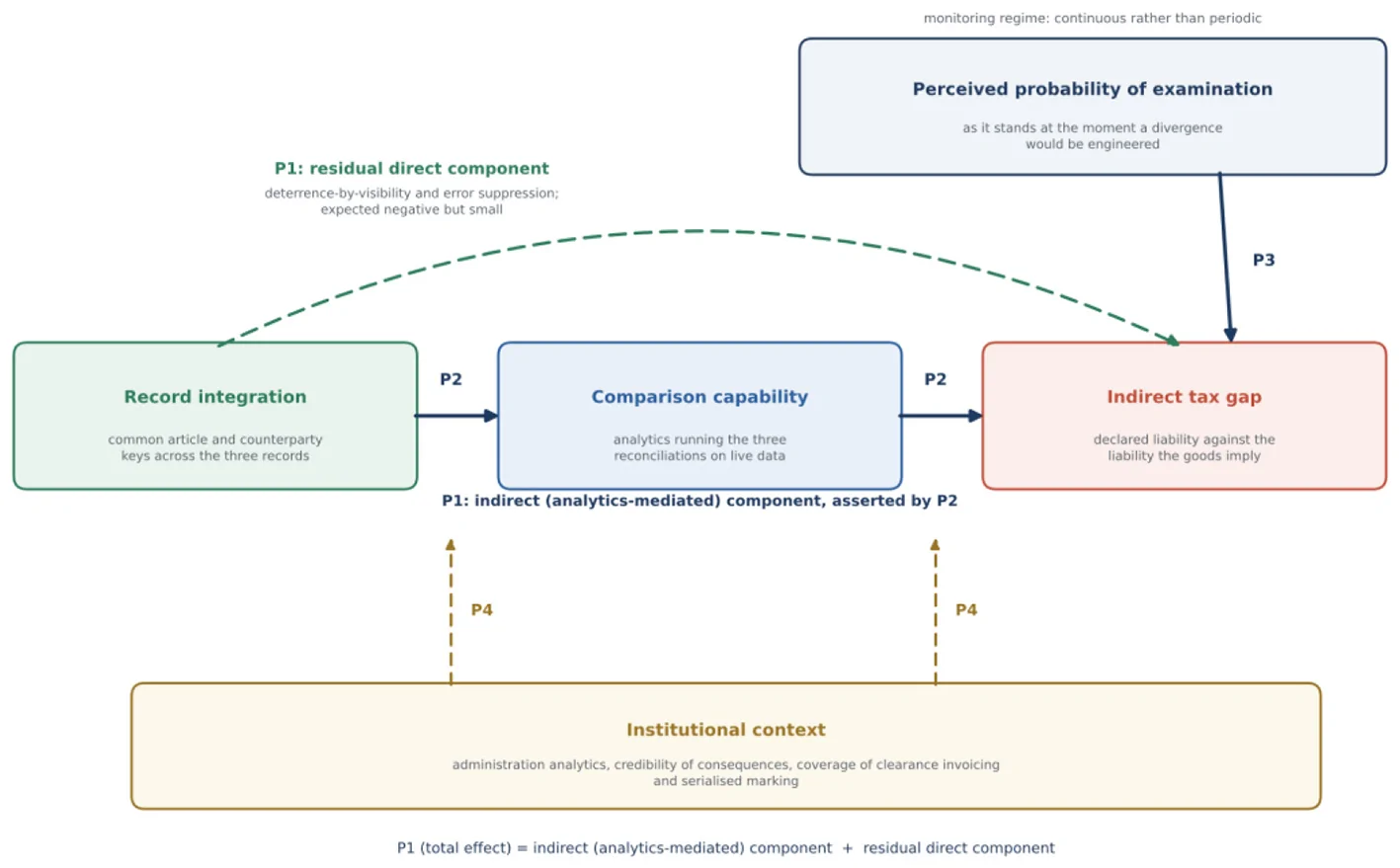
The four propositions as a single model. Record integration acts on the indirect tax gap along two components of the total effect P1: the solid chain through comparison capability, which is the analytics-mediated path asserted by P2, and the dashed path, which is the residual direct effect of integration (deterrence-by-visibility and error suppression), expected negative but small; P1 is the sum of the two. The monitoring regime acts through perceived probability of examination (P3), and institutional context moderates both transmission paths (P4). The expected pattern is complementary partial mediation (Zhao et al., 2010).

Proposition 1 (total effect). The tighter the integration among an enterprise's physical, transactional and fiscal records, the smaller its indirect tax gap. P1 is stated as a total effect: it sums every path from integration to the gap, whether or not analytics are running. Against it would count enterprises with fully integrated records whose measured gaps match those of unintegrated peers once size, sector and enforcement exposure are held fixed.

Proposition 2 (mediation). Integration works mainly through comparison. Analytics capability mediates the effect of record integration on the tax gap: in the decomposition of the total effect, the indirect path running from integration through operating analytics to the gap carries most of the weight, and integration without operating analytics yields little. Against it would count compliance gains that appear upon integration alone, before any detection logic runs, and persist without it. The closest existing evidence is the Rwandan finding that e-invoicing raises payments chiefly by making audits more efficient rather than by making adopters spontaneously compliant (Kotsogiannis et al., 2025). It is consistent with the proposition without testing it at the level of the firm's own records.

Whether P1 is a total effect with P2 as its mediator, or whether a residual direct effect remains once the mediated path is accounted for, deserves a precise answer rather than a diagram's ambiguity. The relationship is this. P1 is the total effect, and P2 asserts that the indirect, analytics-mediated path is its dominant component. The framework predicts a residual direct effect that is negative but small, so the expected pattern is complementary partial mediation in the taxonomy of Zhao et al. (2010), not full mediation. The residual direct path exists for two identifiable reasons. First, deterrence-by-visibility: integration itself changes the decision problem of the person at the keyboard, because linked records make the existence of a future comparison salient even before any comparison runs, and Section 7's mechanism operates on perceived, not actual, examination. Second, error suppression: shared keys and automatic linkage remove a stratum of inadvertent divergence, mismatched articles, double postings, lost documents, that contributes to the measured gap without any evasive intent and disappears without any analytics. Both mechanisms are real but bounded, which is why the framework expects them to carry the smaller share. The prediction is falsifiable in both directions: full mediation (a residual direct effect indistinguishable from zero) would count against the two mechanisms just named, while a dominant direct effect would count against P2 itself. Empirically the decomposition is standard: the total effect is estimated without the mediator, the indirect effect through the product of paths with the mediator included, and the residual direct effect as their difference, with the identification threats listed in Table 6. Figure 4 draws this structure explicitly. The solid paths carry the mediated chain asserted by P2, and the dashed path from record integration to the tax gap is labeled as the residual direct component of P1. P1 as a whole is the sum of the two.

Proposition 3. Holding examination resources fixed, continuous comparison deters more than periodic audit, because it raises perceived detection at the moment of decision rather than at the season of inspection. Against it would count experiments in which units under always-on monitoring declare no differently from units audited annually at equal cost.

Proposition 4. The yield of enterprise capability is set by institutional context. Administration analytics, the credibility of consequences and the coverage of external infrastructure such as clearance invoicing and marking moderate every link above. Against it would count uniform capability effects across jurisdictions that differ sharply in enforcement credibility.

None of the four is tested here. What we can do, and what conceptual work in this area usually omits, is to say what would have to be measured.

### 8.2 Constructs, indicators and data sources

Table 4 specifies each construct, its definition, candidate indicators, the data that would supply them and the level at which they vary. Three points deserve emphasis. The indicators are extractable from systems a firm already has at stage 2 of Section 10, and none requires a survey instrument, which matters because institutional theory warns that self-reported capability is inflated where adoption is ceremonial. The tax gap is the hardest construct, since the quantity wanted is unobservable by construction; the honest options are audit-assessed additional liability, conditional on selection, and administration risk scores, themselves model output. The index of Equation 5 is a candidate, not a validated measure, and should be treated as a formative index requiring work on weights, redundancy and criterion validity (Diamantopoulos and Winklhofer, 2001), using the default and criterion stated in Section 4.2.

**Table 4:** Constructs, indicators and data sources for the propositions of Section 8.1.

Construct	Working definition	Candidate indicators	Data source	Level of variation
Record integration	Extent to which article and counterparty identifiers are shared, and movements linkable, across the three records without manual matching	Share of articles carrying one master key across warehouse, costing, invoicing and return; share of invoice lines automatically linked to a goods movement; share of marked product groups whose codes are bound to the item master	Enterprise system and marking-system extracts; systems audit	Enterprise-period
Comparison capability	Frequency, coverage and closure of the three reconciliations	Reconciliations run per period; share of articles covered; measured false-positive rate; median days from alert to signed disposition	Alert and disposition logs	Enterprise-period
Record agreement	Normalized inverse of the reconciliation residuals, Equation 5	Absolute ε^PT^ scaled by documented movement; absolute ε^TF^ and ε^PF^ scaled by declared tax	Reconciliation output	Enterprise-period-article
Indirect tax gap	Difference between the liability the goods imply and the liability declared	Audit-assessed additional value added tax; administration risk score; bottom-up gap estimate where available	Administrative microdata; audit files	Enterprise-period
Institutional context	Strength of external infrastructure and of consequences	Coverage of clearance invoicing and of serialized marking; audit rate; share of assessments actually collected; presence of automated invoice scoring	Published administration statistics; legal instruments	Jurisdiction-year
Monitoring regime	Whether comparison is continuous or periodic	Assignment indicator	Experimental design	Enterprise

Indicators are candidates for operationalisation, not validated scales. Section 11.3 states the psychometric work that would precede their use.

## 9 Instantiation: industrial enterprises in Uzbekistan

### 9.1 A state that built two of the three records

Uzbekistan makes an unusually clean setting for the argument, because the state has already built two of the three records from outside the firm. Electronic invoicing was introduced by Cabinet of Ministers Resolution No. 522 of 25 June 2019 (Cabinet of Ministers of the Republic of Uzbekistan, 2019a), available voluntarily from 1 July 2019 and compulsory from 1 January 2020. Invoices are created in a structured format on a central platform, signed digitally, and validated by the tax authority before acquiring legal force. This is a clearance model in the strict sense, and it means that the administration holds a population-scale mirror of the transactional record as it is created rather than a summary of it after the fact.

Digital labeling followed on a separate track. Resolution No. 944 of 23 November 2019 (Cabinet of Ministers of the Republic of Uzbekistan, 2019b) launched the phased introduction of identification means, and Resolution No. 737 of 20 November 2020 (Cabinet of Ministers of the Republic of Uzbekistan, 2020a) established the mandatory system for specified goods. Resolution No. 833 of 31 December 2020 (Cabinet of Ministers of the Republic of Uzbekistan, 2020b) replaced No. 737 and set the operating rules for the national information system Asl belgisi. Sectoral instruments followed, among them Resolutions Nos. 148 and 149 of 2 April 2022 (Cabinet of Ministers of the Republic of Uzbekistan, 2022) for household appliances and for medicines and medical devices. The covered groups now include tobacco, alcohol and beer, household appliances, medicines and medical devices, and bottled water and soft drinks. Each unit carries a DataMatrix code combining a product identifier, a randomized serial number and verification data, and every movement from production or import through to retail is reported into the system. For those product groups the state therefore holds a serialized slice of the physical record as well.

Meanwhile the fiscal frame was rebuilt. A new edition of the Tax Code took effect on 1 January 2020 (Tax Code of the Republic of Uzbekistan, 2020). The standard rate of value added tax fell from 20 to 15 percent from 1 October 2019 and from 15 to 12 percent from 1 January 2023. The second cut was announced in the development strategy for 2022 to 2026 and confirmed by the State Tax Committee in September 2022 (State Tax Committee, 2026). Panel A of Figure 5 sets the rate trajectory against the infrastructure milestones. A state that cuts its headline rate by two fifths over 3 years while wiring up transaction-level visibility has made its fiscal strategy explicit: revenue is to come from the integrity of the base rather than the height of the rate.

**Figure 5:**
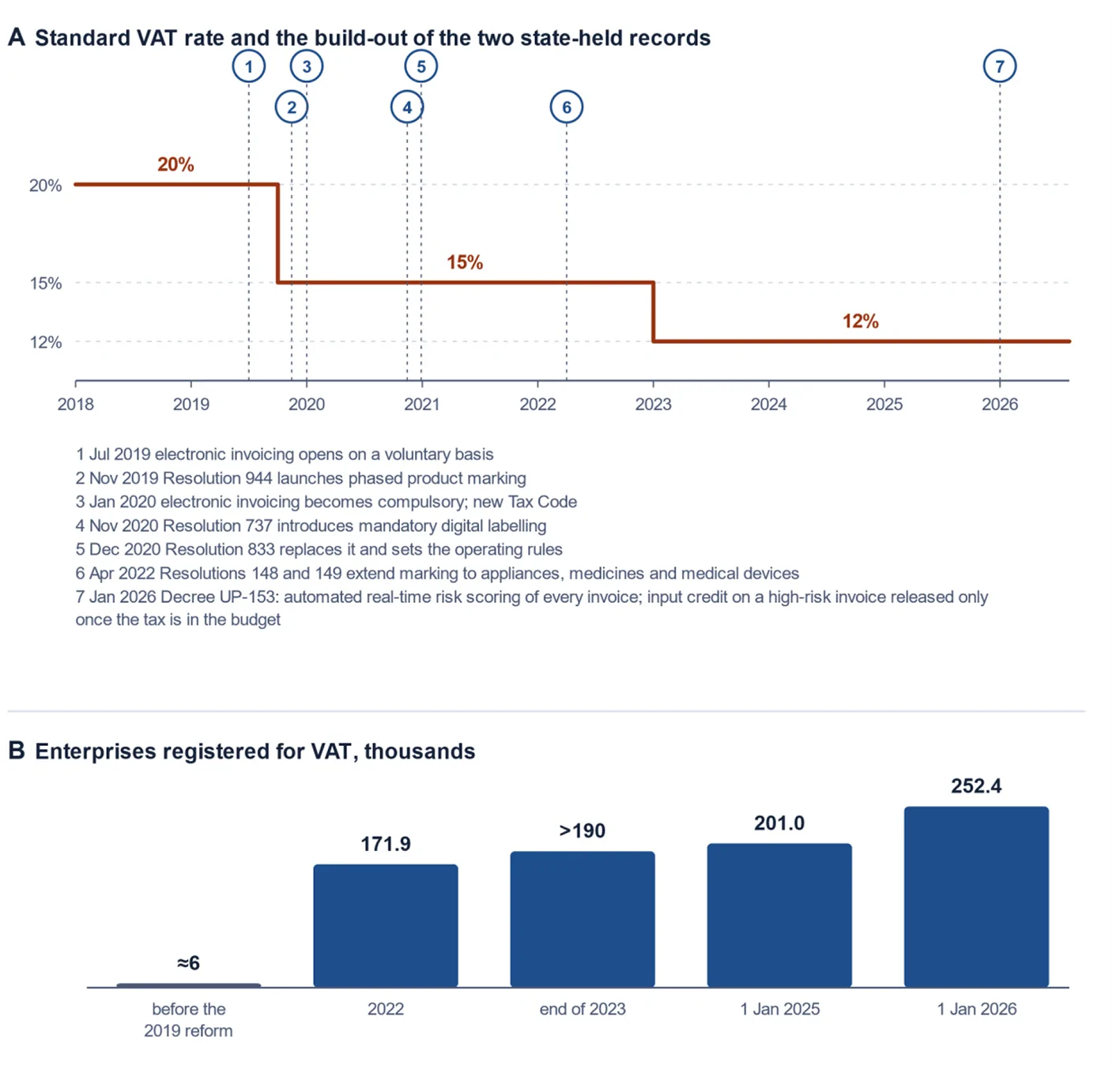
Uzbekistan, 2018 to 2026. **(A)** Standard rate of value added tax against the milestones through which the state built the fiscal record and the serialized part of the physical record. **(B)** Enterprises registered for value added tax, thousands. Sources are listed under Primary legal and institutional sources; the marking track is stated as Resolution No. 944, then No. 737, then No. 833, because No. 833 replaced No. 737 6 weeks after its adoption.

The registration data are consistent with that reading. Official statements report that the number of enterprises registered for value added tax stood at roughly six thousand before the 2019 reform. The State Tax Committee reported 171,900 registered payers for 2022 and more than 190,000 at the end of 2023; 201,023 were registered at 1 January 2025 and more than 252,400 at 1 January 2026 (State Tax Committee, 2026). Panel B of Figure 5 shows the series. A base that has widened by a factor of forty in 7 years is a base whose integrity now depends on what happens inside a very large number of firms that were outside the system a short time ago. The stakes are not small. An independent estimate using the collection-efficiency method puts the compliance component of Uzbekistan's value added tax gap at roughly a quarter of potential revenue on average over 2016 to 2020, with the policy component responsible for a further third of the ideal loss (Niyazmetov, 2023). Whatever the margin of error on such estimates, they place the quantity the framework addresses among the largest fiscal magnitudes in the economy.

### 9.2 The turn of 2026: automated scoring and conditional credit

Two changes taking effect on 1 January 2026 move the setting from an illustration of the framework to a test of it. Under Presidential Decree No. UP-153 (President of the Republic of Uzbekistan, 2026) the administration analyses every electronic invoice in real time and assigns it a risk level, low, medium or high. The assessment is fully automated, without human intervention. The share of a taxpayer's invoices that may be classified high risk in a reporting period is capped at 10 percent. Most consequentially, input tax on an invoice classified high risk is creditable to the buyer only after the tax has been paid to the budget, either by the seller or by the buyer acting as tax agent. The system entered pilot operation on 22 December 2025 with no enforcement action during the test phase. In parallel, from the same date the administration prepares pre-filled returns, including for value added tax.

In the vocabulary of this paper, the administration has automated the transactional-to-fiscal comparison from the outside and has attached a cash consequence to its output. Three implications follow. First, the enterprise's own ε^TF^ check ceases to be a compliance nicety and becomes a working-capital control, because a counterparty's risk classification now determines when the enterprise recovers its own input tax. Second, the 10 percent cap is a rationing device rather than a measurement. A firm's exposure therefore depends on the distribution of risk across its supplier base and not only on its own conduct, and the counterparty analytics of Section 6.1 move from the optional column to the necessary one. Third, and most important for the framework, the automation covers one edge of the triangle and not the other two. The administration can compare documents with declarations at population scale. It cannot compare either with the goods, except for the marked product groups and even there only at the granularity the codes carry. The physical-to-transactional and physical-to-fiscal comparisons remain, as a matter of information structure rather than of policy choice, things only the enterprise can perform. The pre-filled return deserves one further remark in the light of Section 4.4. It is derived from the administration's own cleared-invoice mirror, so reconciling one's return against it tests transcription, not truth. An enterprise that treats that reconciliation as assurance has mistaken an echo for an anchor.

### 9.3 Record agreement in administrative practice: the tax gap coefficient episode, 2021 to 2023

Have actual gaps been identified from the reconciliation between invoices and returns in Uzbekistan, and can the proposed system of checks be illustrated from known practice? They have, and it can, because the administration has already operated a population-scale version of one edge of the triangle, and the episode, documented in the public record, illustrates both the power of the mechanism and the governance failure mode that Section 6.2 warns against.

From 2021 the State Tax Committee ran an automated detection of breaks in the value added tax chain, known in practice as the tax gap (in Uzbek usage, soliq uzilishi) and computed across the cleared-invoice graph. Where value added tax charged along a supply chain was not matched by tax actually received by the budget at some link, the discrepancy was identified and propagated to the taxpayers connected to it. A coefficient derived from these breaks was applied, among other uses, in the processing of refund claims. Two properties of the implementation are documented in contemporaneous reports of the public meetings between the administration, the Ministry of Finance and business representatives in April 2022. First, the break computation reached beyond a taxpayer's immediate counterparty to non-payment by second, third and subsequent links of the chain, so that a compliant firm's refund position depended on the conduct of counterparties it had never contracted with. Second, the criteria were not calibrated by sector, and business representatives proposed at those meetings that collective responsibility be limited to the first-tier counterparty and that the risk criteria reflect industry specifics (Gazeta.uz, 2022; Anhor.uz, 2022). In August 2023 the President instructed that the practice of applying the tax gap coefficient in the refund of overpaid value added tax be abolished. The Chamber of Commerce and Industry subsequently reported that refunds in January to April 2024 amounted to 7.5 trillion soums, against 5.3 and 5.4 trillion for the whole of 2022 and 2023 respectively, and attributed the roughly forty percent rise to the abolition (Spot.uz, 2024).

Read through the framework, the episode carries four lessons, and they support the paper's propositions rather than merely decorating them. First, it demonstrates feasibility: a population-scale, automated ε^TF^-type reconciliation with cash consequences is not a proposal but an operated fact in this jurisdiction, which is why the framework's premise, that the comparisons are now cheap, needs no speculative defense. Second, it instantiates the false-positive discipline of Table 3 and the proportion gate of Figure 3 at national scale. The propagation of breaks through distant tiers was, in the framework's terms, an alert logic whose principal false-positive driver, innocent exposure to remote counterparties, was not modeled. The prediction attached to Table 3, that a technique whose false-positive driver is not modeled will be abandoned by its users, was realized in the strongest available form: withdrawal of the instrument itself. Third, it gives Proposition 4 documented content: the yield of the reconciliation depended not on its detection logic, which was sound in principle, but on the institutional envelope, calibration, contestability and the allocation of liability, in which it ran. Fourth, it explains design features of the 2026 regime that would otherwise look arbitrary. The 10 percent cap on high-risk classifications, the pilot period without enforcement, and the shift from refund-side coefficients to invoice-level conditional credit read naturally as institutional learning from the episode, embedding proportionality where the earlier instrument lacked it. The episode also disciplines the paper's advice to enterprises. Under either regime the firm's rational response is the same: run the transactional-to-fiscal comparison on itself, with counterparty analytics, before the administration runs its version. That is precisely the capability Sections 6 and 10 specify and that Section 9.4 grounds in the software actually in use.

### 9.4 Implementing the reconciliations in the enterprise software in daily use

How do the framework and its indicators integrate with the software enterprises actually run, and what should enterprises concretely do under e-invoicing with automated risk scoring? The question has a definite answer in this jurisdiction, because the software landscape is unusually homogeneous. Accounting and enterprise management in Uzbekistan is dominated by systems of the 1C:Enterprise family. The connection between those systems and the state platform is intermediated by authorized electronic document interchange operators, a register maintained by the Tax Committee that had grown to some 27 organizations by 2025, of which Didox and Faktura.uz are prominent examples. The operators provide modules and interfaces through which invoices are signed and transmitted from within the accounting system, incoming cleared documents are synchronized back into it, and, for marked goods, the Asl belgisi marking system is reached (CRPT-Turon, 2024; Norma.uz, 2021). The three reconciliations of Figure 1 therefore do not require new infrastructure; they require configuring comparisons across data objects that a typical enterprise already holds. Table 5 states the mapping.

**Table 5:** Implementing the three reconciliations, and the counterparty control that the 2026 regime makes necessary, in the enterprise software in daily use in Uzbekistan.

Reconciliation edge	Data objects in a 1C-class enterprise system	External anchor (Section 4.4)	Control to configure, and its owner
ε^PT^: physical to transactional	Warehouse (stock) registers; goods receipt and issue documents; production output reports; write-off acts	Serialized marking scans reported to Asl belgisi; weighbridge and meter feeds; independent cycle counts	Article-level stock identity, Equation 1, run each period per article; every movement blocked without a document; yield monitored against engineering tolerance with dispersion tracked over time. Owner: operations controller.
ε^TF^: transactional to fiscal	Sales and purchase invoice journals; VAT ledgers; the return preparation module	The administration's cleared-invoice mirror via the operator interface and the taxpayer cabinet; the counterparty's copy	Line-by-line tie-out of ledgers to cleared invoices and to the return before filing; automatic matching of every input credit to a cleared invoice of a live counterparty; cut-off report of document dates against period boundaries. Owner: chief accountant.
ε^PF^: physical to fiscal	Write-off and disposal acts; stocktake results; rate and exemption assignment in the item master	Marking-system exit statuses for serialized goods; destruction evidence with independent timestamps; inspection calendar	Losses declared for tax matched to documented destruction; write-off timing screened against stocktake and inspection dates; rate and exemption categories audited against the goods physically present. Owner: tax manager.
Counterparty risk (supporting ε-TF under automated scoring)	Supplier master data; contract and payment records	Registry status, registration age and risk classification of counterparties as visible through the state platform	Pre-transaction counterparty screening at onboarding and at each order; continuous monitoring of the share of incoming invoices classified high risk against the 10 percent cap; credit on a high-risk invoice not booked as recoverable until payment to the budget is confirmed. Owner: procurement together with the chief accountant.

The data objects named are generic to 1C-class systems; the anchors are the components that satisfy the independence condition of Section 4.4; and every control ends, per Figure 3, in a disposition signed by its named owner.

Three pieces of advice for enterprises follow from the table and from the 2026 regime, stated in the imperative. Run the transactional-to-fiscal comparison on yourself before the administration runs its version: the marginal cost inside an operator-connected 1C-class system is close to zero, and the consequence of not doing so, after Decree UP-153, is measured in working capital. Treat counterparty analytics as a procurement function, not an accounting afterthought. The conditional-credit rule makes the risk distribution of the supplier base a direct determinant of the firm's own cash position, and the 10 percent cap makes the share of high-risk incoming invoices a quantity to be managed, not observed. And bind the anchors before buying the analytics. DataMatrix codes tied to the item master, meters wired into the yield comparison and cycle counts under separate ownership are the components that make the other two edges evidential at all, for the reasons Section 4.4 states. A firm that skips them will reconcile records against their own copies and find no discrepancies.

### 9.5 What the state cannot build

Only the enterprise can align its item master with the codes on its goods, connect warehouse movements to invoice lines, and run the three comparisons on itself before the administration runs its version of one. Where that internal work is skipped, institutional theory predicts ceremonial operation, present in form and absent in function (DiMaggio and Powell, 1983; Meyer and Rowan, 1977). Cross-country evidence agrees that mandated tools pay off only where uptake is real (Bellon et al., 2022; Hesami et al., 2024). The setting therefore supplies Proposition 4 with a natural test population of enterprises facing identical mandates, and Proposition 3 with an opportunity, since the December 2025 pilot without enforcement and the enforcement period that followed differ in exactly the dimension the proposition concerns.

### 9.6 Boundary conditions

Three conditions bound the transfer of the argument, and they are conditions rather than caveats because the framework's predictions change across them. Process industries, where mass balances are tight and θ^PT^ is naturally small, suit continuity testing better than job shops, where the arithmetic loosens and the sustainable gap of Equation 7 is correspondingly larger for the same investment. Small enterprises carry the fixed costs of integration badly, and for them shared platforms or administration-supplied analytics may have to substitute for in-house capability, a substitution the optimal administration literature would count as enforcement expenditure (Keen and Slemrod, 2017). And where master data are poor, analytics faithfully amplify the defects of their inputs, so remediation precedes sophistication; an enterprise whose item master contains duplicate articles will generate residuals that are artifacts of its own data and will learn nothing from them.

## 10 A maturity path for enterprises

The account implies a definite order of construction, set out in Figure 6 as four stages with their markers and traps. An enterprise first makes its separate records honest, cleaning the item master, closing the gap between counted and recorded stock, and rebuilding one tax period from primary documents to learn where its triangle breaks. In the language of Section 4.4, this is where the anchors are bound. It then connects the records, beginning with the product families where fiscal exposure concentrates. Only then does comparison begin, piloted on one site for one full tax cycle with measured false-positive rates, and only after that does it become continuous.

**Figure 6:**
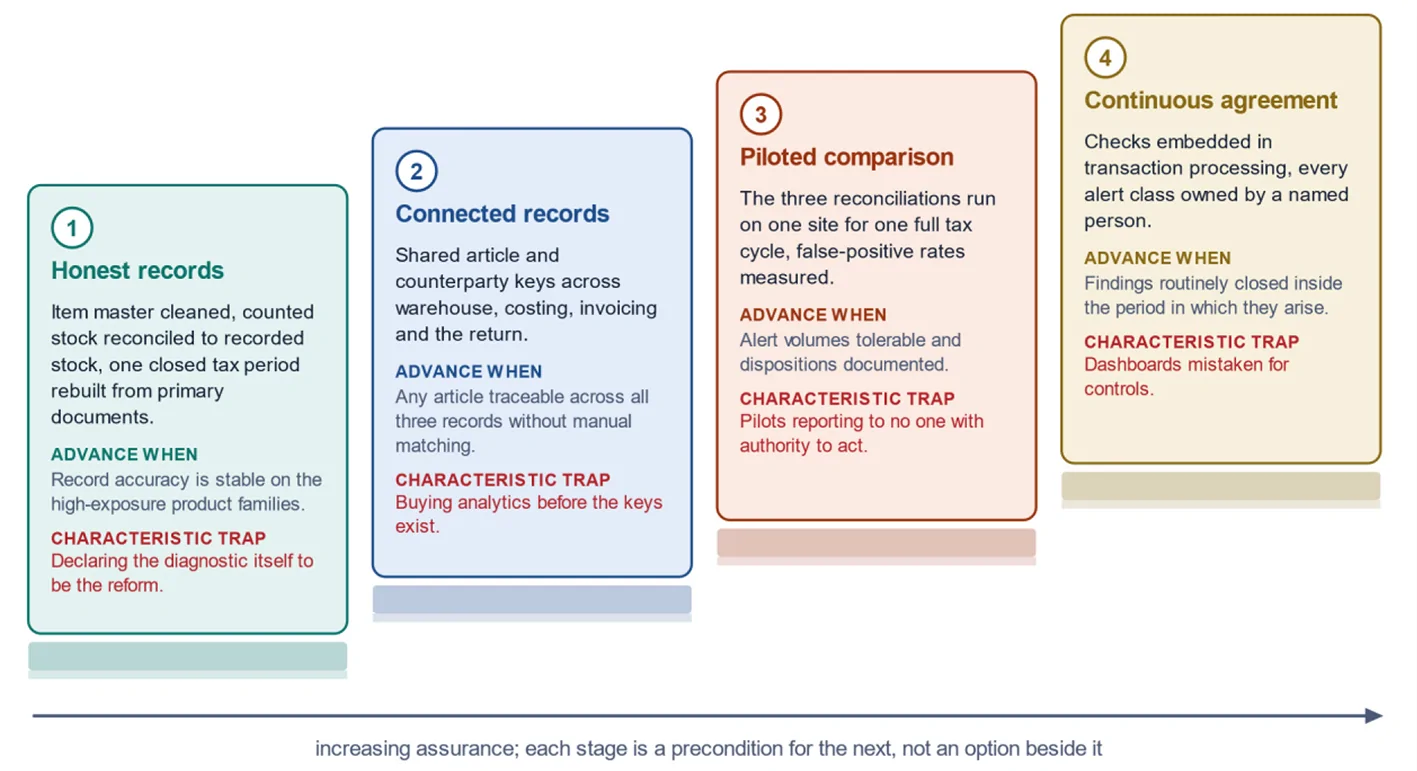
Four stages toward continuous record agreement, with the marker that licenses advancing and the trap characteristic of each stage.

The sequence applies the dynamic capabilities tradition to fiscal capability (Teece et al., 1997; Teece, 2007), and its two recurring failures mirror its two middle stages: analytics bought before the records deserve them, and pilots whose findings no one is empowered to act on. The audit society's pathology, checking for the sake of being seen to check (Power, 1997), is the terminal form of both, and the defense is the same: every comparison must end in a disposition signed by a person.

## 11 Discussion

### 11.1 Contribution

The contribution begins with a change of unit. Where compliance economics analyses the reporting decision and administration research the state's enforcement production, the account here analyses the relations among an enterprise's own records, and locates accountability in the testable agreement of those relations under stated independence conditions. The change does concrete work. It explains why record-by-record audits certify schemes rather than catch them, and converts the four leakage patterns into engineered divergences, each with a detector and a residual. It imports the continuity architecture of continuous assurance (Kogan et al., 2014) into taxation, where its equations hold as identities, and gives the deterrence tradition a within-firm mechanism administrations cannot produce from outside.

Section 4.5 adds a second contribution. Equation 7, now derived from four stated assumptions rather than asserted, makes the sustainable gap the sum of the binding measurement tolerances rather than a function of the number of controls operated. It derives the rule that investment belongs at the weakest comparison, explains why control inflation is common and useless, and predicts that instrumenting a previously unseen product family cuts the gap discontinuously while refining an already binding measurement does not. The sensitivity analysis extends this: relaxing separability makes the bound a lower envelope rising with every coarsening of the item master, relaxing exogenous tolerances turns dispersion into an object of monitoring, and relaxing zero detection prices risk-tolerant evasion in expected periods-to-detection. These are comparative statics and they can be wrong, which is the point of stating them.

The account also carries a benefit that softens its cost: the triangle that protects the tax base protects the firm against shrinkage, procurement fraud and costing error, so the investment is an internal-control upgrade that happens to make declarations verifiable.

### 11.2 Implications for enterprises and for administrations

For enterprises the implication is an order of work rather than a shopping list, stated in Sections 9.4 and 10 down to the modules in daily use. For administrations it is less obvious. Automating one edge, as Uzbekistan has done for the transactional-to-fiscal comparison, buys wide coverage at low marginal cost and simultaneously raises the return to enterprise capability on the other two, because the channels it cannot see are the ones that survive. The prediction is specific: in a clearance-model jurisdiction with automated scoring, the residual gap in manufacturing should concentrate in unrecorded output and disguised disposal, more sharply as invoice-level controls tighten. This is a testable displacement claim in the tradition of Carrillo et al. (2017) and Slemrod et al. (2017), applied inside the firm.

What accountability measures and cross-checks can the state further improve? The framework, read together with the episode of Section 9.3, yields a concrete list. First, publish the operating characteristics of the scoring system, above all its false-positive rate and the distribution of classifications by sector. A tolerance that is a published property disciplines behavior on both sides, which is the mechanism of Section 7 applied to the administration itself. Second, keep liability graduated and proximate: the documented failure of multi-tier collective responsibility argues for consequences that attach to the first-tier relationship a firm can actually monitor, with remoter breaks pursued against their authors. Third, calibrate risk criteria by sector, since Table 3's false-positive drivers are sectoral facts, and an uncalibrated screen taxes honest heterogeneity. Fourth, open an explainability and appeal channel for high-risk classifications, so that the ownership gate of Figure 3, a named owner and a recorded disposition for every alert, operates on the administration's alerts as it should on the firm's. Fifth, extend the automated cross-checks to the physical edge where the state already holds the data. For marked product groups, the marking system's exit statuses can be reconciled against cleared invoices at population scale. This closes on disguised disposal, which is precisely the channel the invoice-level controls displace evasion toward. Sixth, expose the firm-side data through interfaces that let enterprises pull their own risk position, counterparty classifications and break history. Section 9.3's central lesson is that the reconciliation performs best when the taxpayer can run it on itself before the administration runs its own version with cash consequences attached.

### 11.3 Limitations

The claims are argued, not demonstrated, and the propositions await data. The literatures synthesized lean toward large firms in high-income settings, so transfer to small enterprises and transition economies is itself among the hypotheses. The behavioral core is deterrence, and richer accounts of tax morale would extend rather than contradict it: staff who comply because they think compliance right leave the same residuals as staff who expect to be caught, and the framework cannot distinguish them.

Two earlier limitations are now results rather than caveats: the independence problem raised by integrated systems is an explicit condition (Section 4.4), and the behavior of Equation 7 under relaxed assumptions is analyzed rather than conceded (Section 4.5). Three remain specific to the formalism. Equation 5 is still an index without established measurement properties; its structure and weighting default are justified and its validation route specified, but the psychometric work remains. The endogeneity of tolerances is analyzed directionally, not modeled structurally; a full treatment would make θ a strategic variable in a game between designer and monitor. And the valuation channel is closed only partly, for the reason given in Section 5.4. The artifacts of Figures 1, 3, 6 are planning instruments offered at the design science stage of argued utility (Hevner et al., 2004).

### 11.4 A research programme

Table 6 turns the propositions into designs and names the identification threat most likely to defeat each. The first two call for archival designs on the administrative microdata that clearance invoicing and marking now generate, joined where access allows to enterprise records, with the mediation decomposition of Section 8.1 estimated in the standard total, indirect and residual-direct form (Zhao et al., 2010). The third calls for field experiments in the lineage of randomized enforcement research (Naritomi, 2019; Pomeranz, 2015). The pilot-then-enforcement sequence of Section 9.2 approximates it naturally, and the abolition of the tax gap coefficient in Section 9.3 offers a second natural experiment on the institutional margin. The fourth calls for comparative work across jurisdictions and longitudinal tracking up the stages of Figure 6 (Teece et al., 1997). Alongside all four runs a design science track: implementing the artifacts with partner enterprises through build-and-evaluate cycles (Hevner et al., 2004), including the psychometric work any indicator set needs. No single method carries a claim spanning behavior, systems and institutions, so the programme is deliberately plural (Snyder, 2019).

**Table 6:** From propositions to research designs, with the principal identification threat to each.

Proposition	Claim in brief	Design suited to it	Primary data	Principal identification threat
P1	Record integration is associated with a smaller tax gap	Cross-enterprise archival comparison with sector, size and enforcement controls	Invoice clearance microdata; marking records; enterprise systems	Selection: capable firms may integrate and comply for the same unobserved reason
P2	Comparison capability mediates the effect of integration	Mediation analysis on staged adoption; difference-in-differences around module go-live	Capability audits paired with declaration histories	Simultaneity: integration and analytics are often purchased together
P3	Continuous comparison beats periodic audit at equal cost	Randomized assignment of monitoring regimes; pilot-then-enforcement discontinuities	Declaration behavior; alert and disposition logs	Spillovers between treated and control units through shared suppliers
P4	Institutional context moderates every link	Multi-jurisdiction comparison; longitudinal capability tracking	Cross-country administrative data; repeated enterprise observation	Jurisdictions differ on many dimensions at once; few effective degrees of freedom

## 12 Conclusion

This article asked how digital technologies and big data can strengthen indirect tax accountability in the inventory accounting and auditing of industrial enterprises, and answered with a change of perspective rather than a catalog of tools. Goods leave three records as they move through a firm, and taxation is honest exactly where those records agree, provided they are anchored in mechanisms a single keyboard cannot reach. Evasion is quiet divergence and assurance is organized comparison; digital infrastructure matters because it makes comparison continuous and cheap. Formally, the gap an enterprise can sustain equals the sum of the measurement tolerances of the comparisons that bind, a stronger claim than the observation that more data help, and one that now travels with its assumptions and their sensitivity.

Uzbekistan gives the argument its urgency. A state that has cut its headline rate while mandating cleared invoices, serialized marking and, from 2026, automated risk scoring with conditional input credit has bet its revenue on the integrity of the base, and that base runs through the warehouses. The country has also lived through one full cycle of population-scale reconciliation, from deployment through contestation to redesign, and that documented experience is now part of the argument. The infrastructure for the fiscal record and part of the physical record exists. Whether triangles form on top of it will be decided inside enterprises, in item masters, event logs and the unglamorous work of reconciliation. The four propositions say what should be observed if the account is right and what would show it wrong.
